# Impact of the SARS-CoV-2/COVID-19 pandemic on the patient journeys of those with a newly diagnosed paediatric brain tumour in the UK: a qualitative study

**DOI:** 10.1136/bmjopen-2024-086118

**Published:** 2025-01-02

**Authors:** Kalsoom Akhter, Roland Casson, Liz Brewster, G A Amos Burke, John-Paul Kilday, Donald Macarthur, Rachel Isba, Ibrahim Jalloh

**Affiliations:** 1 Cambridge University Hospitals NHS Foundation Trust, Cambridge, UK; 2 Lancaster University, Lancaster, UK; 3 University of Birmingham, Birmingham, UK; 4 Central Manchester University Hospitals NHS Foundation Trust, Manchester, UK; 5 Nottingham University Hospitals NHS Trust, Nottingham, UK; 6 Alder Hey Children's NHS Foundation Trust, Liverpool, Merseyside, UK

**Keywords:** COVID-19, Paediatric oncology, PAEDIATRICS, Health Services

## Abstract

**Abstract:**

**Objectives:**

To explore the impact of the SARS-CoV-2/COVID-19 pandemic on the diagnosis, management and patient journey for children and young people with a newly diagnosed brain tumour in the UK.

**Design:**

Exploratory qualitative study focused on patient journeys from multiple perspectives, conducted as part of a wider mixed-methods study.

**Setting:**

Three paediatric oncology tertiary centres in the UK.

**Participants:**

10 children and young people with brain tumours (n=6 females, n=4 males), 20 caregivers (n=16 females, n=4 males) and 16 stakeholders (specialist nurses, consultant neurosurgeons and oncologists, and representatives from brain tumour charities) were interviewed between January 2022 and June 2023.

**Results:**

The paper incorporates multiple perspectives, including those of children and young people, parents/caregivers, clinical staff and charity representatives, to explore the patient journey. Five themes describe the journey for new patients with paediatric brain tumour during the pandemic, focusing on (1) challenges getting into the healthcare system, (2) managing as a family during restrictions imposed by the pandemic, (3) complexities of building a cohesive and supportive healthcare team, (4) difficulties caregivers experienced in accessing practical and emotional support in hospital and (5) ongoing difficulties experienced by families in the community.

**Conclusions:**

Findings from this study offer practical insights from children, parents/caregivers and relevant stakeholders to improve the healthcare system during future disruptions. Overall, this study not only sheds light on the challenges faced by families during the pandemic but also provides suggestions for improving healthcare services to ensure a more comprehensive and effective response in times of crisis.

**STRENGTHS AND LIMITATIONS OF THIS STUDY**

- We collected rich data that incorporates multiple perspectives, including those of children and young people, caregivers, clinical staff and charities from different regions of the UK.
- A limitation is that participants were self-selecting and we were unable to recruit any bereaved families.
- The retrospective nature of the study posed challenges, particularly for children and young people recalling experiences.

## Background

 The global SARS-CoV-2/COVID-19 pandemic, declared by the WHO on 11 March 2020, presented a significant challenge to the provision of healthcare services. In the UK, as in other countries, this impacted the diagnosis and treatment of non-COVID-19 conditions. Evidence suggests that children and young people were less acutely affected by COVID-19 in terms of morbidity and mortality, but that their lives were disrupted in other ways, including access to routine healthcare services. Evidence suggests that there were changing patterns of use as services were impacted by the measures put in place to mitigate the spread of infection.^1^,^4^

This paper investigates the impact of the pandemic on one non-COVID-19 condition: paediatric brain tumours, the most common childhood solid tumour. Every year around 500 children and young people are newly diagnosed with a brain tumour in the UK.^5^ Mortality rates vary according to tumour type, but are generally high, with a 5-year survival rate of 66% overall for all types of brain tumour in Europe.^6^ Around 60% of patients are left with some form of lifelong neurological disability.^7^ Delays in diagnosis can make treatment more complex and increase the likelihood of tumour progression, death or disability, as well as impacting relationships between families and healthcare teams.^8^

Diagnosis is often difficult as symptoms and signs are often non-specific. Initial symptoms are often picked up in optometry, primary care, emergency departments or in nurseries and schools, with research suggesting that in around 40% of cases, initial detection of paediatric brain tumours occurs in optometry.^7^ Care for paediatric brain tumours is complex, and treatment and rehabilitation require strong interdisciplinary and interagency collaboration across hospital and community-based health, education and social care services.^8^ Recent research into family experiences of paediatric brain tumours has concluded that the psychosocial needs of children, young people and families need to be prioritised.^9 10^ There is some emerging evidence that the relationships between families and healthcare staff were disrupted during the pandemic^11^ and that the experience of being hospitalised with a condition (such as a brain tumour) that necessitates careful infection prevention measures is isolating.^12^

As part of a wider study exploring the diagnosis, management, clinical outcomes and patient/carer experiences of receiving treatment for a paediatric brain tumour during the pandemic, we sought to answer the research question: What is the impact of the COVID-19 pandemic on the diagnosis, management and patient journey for children and young people with a newly diagnosed brain tumour in the UK? By exploring the impact of the pandemic on the patient journey of those diagnosed with a paediatric brain tumour at the time, the paper presents internationally relevant lessons about how healthcare services may need to prioritise maintaining particular services to prevent delays in the diagnosis of childhood cancers and ensure better outcomes for children and young people.

## Methods

Interviews were used to collect detailed qualitative data about the experiences of children and young people, parents/caregivers, clinical staff working in hospitals and representatives of paediatric brain tumour charities at three paediatric oncology centres in the UK. The qualitative approach allowed us to explore participants’ understanding of their experiences of tumour diagnosis, treatment and care. This was contrasted with quantitative data on clinical outcomes collected as part of the wider mixed-methods study, presented elsewhere. Analysis was conducted using a six-phase reflexive thematic analysis.^13^ We adopted a broadly realist epistemological stance.^14^ By including multiple participant groups, who were based in different hospitals, and having multiple experienced researchers working on a detailed analysis process, we were able to triangulate our findings to ensure they were robust and rigorous.^15^ As researchers, we recognise that meaning is constructed through dialogue and that our values, interests and assumptions shape the research questions and analytical process.

### Recruitment and participants

We recruited participants from three tertiary centres treating patients with paediatric brain tumour. There were two groups of participants: children and young people and their caregivers, and key stakeholders (clinical and allied health professional staff, charities), who provided insights into how treatment and care services may have been disrupted during the pandemic.

We identified eligible patient and caregiver participants through hospital databases. Potential participants were approached if they were diagnosed in the study period and the 12 months prior to the pandemic (ie, 1 March 2019 to 28 February 2021). Caregivers were provided with information about the study by post or by a clinician known to them, and invited to contact the research team if they wished to participate themselves and/or were happy for their child to participate. We approached bereaved families as well as those with surviving children.

Families that declined to participate gave various reasons, including a reluctance to revisit traumatic experiences, other commitments, dissatisfaction with existing services and significant life events affecting their family. All bereaved families declined to participate. Clinical and charity staff were recruited through their organisations, provided with information via email or in team meetings, and were asked to contact the research team if they were able to participate.

We estimated our sample size by referring to similar qualitative studies, and then assessed the adequacy of our sample size during the data collection process, guided by the information power framework.^16^ Overall, according to the information power framework, our sample size was sufficient for developing new insights in line with the study’s objectives.

### Data collection

The research team consisted of two research-active clinicians (IJ, RI), one health psychology researcher (KA), one clinical psychologist (RC) and one medical sociologist (LB). Both interviewers (KA and RC) had extensive experience of working within NHS services, but were independent of the clinical services from which participants were recruited. Interviews with stakeholders were conducted between January 2022 and February 2023 by KA. Interviews with caregivers and children were conducted between May 2022 and June 2023 by KA and RC. Interviews took place 24–44 months after the initial diagnosis.

For children, young people and parents/caregivers, we conducted semi-structured interviews, based on an interview schedule (online supplemental file 1). These typically lasted around 1 hour and were conducted in person or online, depending on participants’ preferences. In interviews with children and young people, for whom recalling treatment experiences was challenging, tools such as Talking Mats and children’s creative work (eg, art, photos) were used to facilitate the interview process.^17^ As a team, we were conscious that interviews might be challenging for participants in terms of recalling traumatic experiences. This was mitigated by the involvement of a clinical psychologist and the clarity that consent could be withdrawn at any stage.

For stakeholders who had been working with patients with paediatric brain tumour during the study period, including clinical staff working in the paediatric neuro-oncology departments of each treating centre, and representatives of paediatric brain tumour charities, we conducted semi-structured interviews, again based on an interview schedule (online supplemental file 2). These interviews typically lasted 30–60 min and were conducted online. All interviews were recorded using a digital voice recorder and transcribed using the online Happy Scribe transcription service.

### Patient and public involvement

Prior to the research study, we conducted a patient and public involvement co-design event with children and families. This event included presentations from the research team and a group discussion. We incorporated feedback into the design in terms of (1) adopting a flexible and inclusive approach to data collection (eg, inviting children to contribute written submissions, drawing or other media as an alternative to interviews) to enable all children to participate and (2) broadening the scope of the interviews to include the patient journey from initial awareness to longer-term adjustment.

Written informed consent was given by parents for all children participating, and informed assent was also given by children and young people aged 11–16. For all other participants (eg, key stakeholders and parents), written informed consent was also given.

### Data analysis

We completed a six-phase reflexive thematic analysis, following Braun and Clarke’s (2022) framework.^13^ First, we immersed ourselves in the data by re-listening to interviews to ensure accurate transcripts, and creating case summaries. Second, KA and RC coded the interview transcripts and then collated interview extracts. Through team discussion, including LB, we generated ideas about parallel experiences for stakeholders and families. Conducting a second round of coding developed more abstract codes, making the large data set more manageable. RC then generated preliminary themes via mind maps, reflective writing, reading, consultation with experts possessing relevant professional and/or lived experience, and regular discussion sessions with LB.

During this phase, we refined and defined these themes. This approach enabled us to explore various ways of interpreting the data, structuring themes and to consider perspectives that may have been missing from the data; for example, those of parents who were bereaved or stakeholders who had left their service. We refined and structured the analysis around the concept of ‘patient journeys’ and aimed to acknowledge the complexities of the family, services and wider systems around the child.

## Results

**Figure 1:**
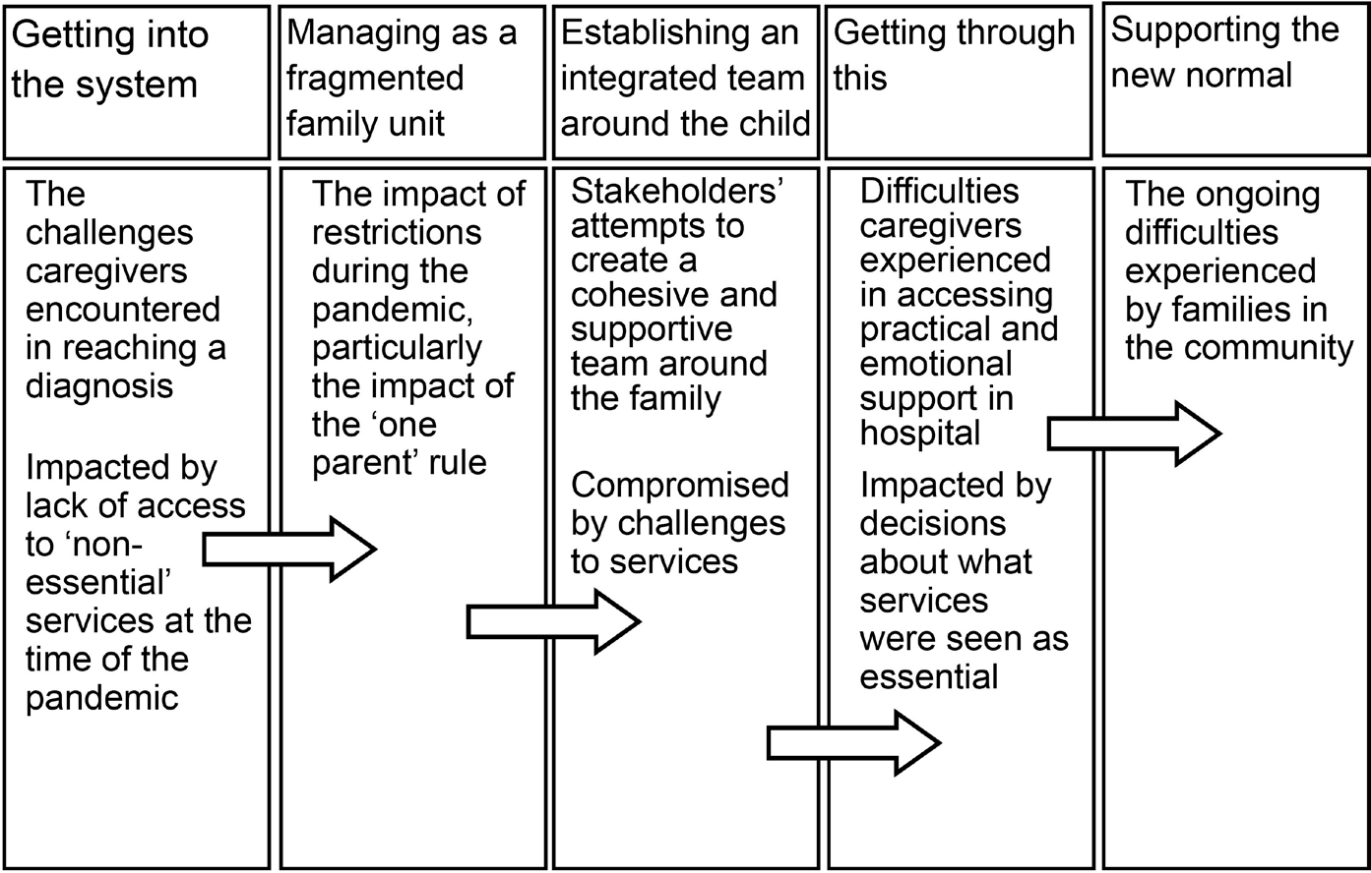
Summary of themes from data analysis.

The paper explores the patient journey from multiple perspectives, including the experiences of children, their families, those delivering healthcare services and charities that supported families at the time. Five themes describe the journey of new patients with paediatric brain tumour during the pandemic (figure 1). First, we describe ‘getting into the system’, the challenges caregivers encountered in reaching a diagnosis and how this was impacted by the lack of access to ‘non-essential’ services at the time of the pandemic. Second, we explore ‘managing as a fragmented family unit’, the impact of restrictions during the pandemic, particularly the impact of the ‘one parent’ rule. Third, we examine ‘establishing an integrated team around the child,’ or how stakeholders’ attempts to create a cohesive and supportive team around the family were compromised by challenges to services. Fourth, we highlight ‘getting through this’, addressing the difficulties caregivers experienced in accessing practical and emotional support in the hospital and how this was impacted by decisions about what services were seen as essential and which were not. Fifth, we address ‘supporting the new normal’, considering the ongoing difficulties experienced by families in the community.

### Participant characteristics

Interview participants were families and key stakeholders based in three tertiary centres, and representatives from national charities. We spoke to 20 caregivers (n=16 females, n=4 males) and 10 children (n=6 females, n=4 males; age range: 5–14 years old at the time of interview). These spanned 18 different family/household units. Age at diagnosis ranged from 4 months to 13 years (mean=7 years), and diagnoses included low-grade glioma, ependymoma, craniopharyngioma and choroid plexus carcinomas.

The 16 stakeholders working within paediatric neuro-oncology included 6 specialist nurses, 6 consultants (neurosurgeons, oncologists), 1 allied health professional and 3 representatives from brain tumour charities.

### Getting into the system

Many families described a prolonged journey from initial awareness to diagnosis, typically encountering multiple attempts by healthcare professionals to reassure and normalise symptoms.

We get delayed diagnosis all the time with brain tumours, because the symptoms are not very specific. So it might be headaches or vomiting or if it’s visual impairment that’s difficult to pick up at the best of times. (Specialist Nurse #4)

This delay was not unique to the pandemic, but the ‘lockdown’ period introduced additional challenges for concerned parents. Public health messages emphasising the importance of protecting the healthcare system led to a reluctance to seek help. Seeing a general practitioner (GP)/ family doctor face-to-face was more difficult and remote consultations relied on clear caregiver reporting to ensure that ‘red flags’ were noted.

I think just by the nature of the pandemic, just as a society, everybody did not want to utilise the NHS unless they absolutely had to. […] You have to weigh up whether it is worth [it]… if somebody is ill enough to take them in. (Caregiver #1)

A lot of them were telephone consultations. They wouldn’t actually see us because of COVID. […] If you explain stuff over the phone, they’re just agreeing with you. They’re just taking your point of view. (Caregiver #17)

Children were also not being seen in other settings such as nursery, school or social situations, making it harder for caregivers to evaluate their concerns.

I think we had a couple of delayed presentations just because they had no idea that their child was different to anyone else. And it wasn't until they became quite sick, because that was then picked up when they brought the child to A&E. (Specialist Nurse #5)

Although some felt that getting onto a treatment pathway during the pandemic was the key challenge, as noted by this specialist nurse, many parents/caregivers emphasised that treatment was compromised throughout the whole patient journey.

### Managing as a fragmented family unit

The national infection control restrictions, which only allowed one primary caregiver to attend hospital with their child, posed significant challenges for families, including siblings and grandparents. Caregivers commented that coming to terms with the diagnosis, managing treatment, collaborating with healthcare teams and supporting each other required togetherness, which was often not possible.

COVID just made it more difficult because it was harder to see people. […] You were in on your own a lot of the time, because some of the time me and [partner] swapped, so we didn’t really communicate much. […] When [daughter] came out of theatre, I wasn't allowed to go and see her, because I wasn’t the designated parent. And it is heartbreaking, absolutely heartbreaking, to not be able to go and see that your child is okay. (Caregiver #15)

We found that the biggest overriding challenge was that many hospitals only allowed one parent carer to accompany a child, which meant that many children and their parents felt isolated from their own family as well. (Charity #3)

Although appreciating the need to manage the risks of infection from COVID-19, families and stakeholders felt that the rules were too rigid. Rules were also applied inconsistently, sometimes differing between families, wards and hospitals, and it was unclear to outsiders why exceptions were made, leading to resentment. On a practical note, opportunities for caregivers to eat, drink, rest and speak to friends and family were severely restricted as they often felt unable to leave the bedside. This was compounded by a lack of access to communal spaces in hospital, and limited activities for children and young people.

For [husband], the whole thing was just awful. […] nobody really explained anything to him […] he was just left a lot of the time on his own. There was just very little support and that’s what I think made it really hard for him. […] sometimes they would bring [child’s] food and then forget about him - because he couldn’t leave the room to get his food. (Caregiver #15)

Establishing relationships and communicating with healthcare teams was challenging because of mask wearing and maintaining social distancing. Comprehending and retaining complex and emotional information without wider support was difficult, and it was harder to involve those outside the room because of technical issues (eg, poor Wi-Fi/mobile phone signal coverage).

You’re not in the right frame of mind to ask those questions because […] them saying ‘it’s a tumour’and you’re [saying to yourself] ‘right’. Then you’ve got oncology coming to see you […] There’s a lot of different emotions you go through, to be honest, which really wasn’t helped by the fact that you can’t all be together as a family. (Caregiver # 13)

Without access to their own practical and emotional support systems, caregivers were conscious of placing additional pressure on depleted healthcare teams.

### Establishing an integrated team around the child

Healthcare workers also experienced significant challenges, including managing uncertainty and confusion, dealing with an increased workload, a sense of guilt and anxiety about assuming unfamiliar roles, and the social isolation inherent in their role during the pandemic.

We don’t want to let our families down. So we were all working extra hours to make sure that things weren’t getting missed and that things were getting done as they should be. None of us wanted the patients and the families to suffer because we were being pulled right, left and centre. (Nurse Specialist #1)

On top of these challenges, it was clear that communication and collaboration within and between teams suffered, impacting on families. Caregivers often received conflicting information, finding out about issues accidentally, referred to as ‘news that leaked out*’* by one parent, or found themselves communicating key information between healthcare professionals. Investigations or treatments were frequently postponed as key people or resources were not available. Clinical services and charities that were deemed ‘non-essential’ by healthcare authorities became less visible, impacting on relationships that would usually be built with families in hospital. This was difficult for all involved.

I had to tell them that it was an incurable brain tumour whilst they were on the COVID ward and that was really difficult because you know you cannot see the family’s faces, they cannot see you and you are telling them that their child is dying. (Consultant #2)

[Families] said they would have liked a conversation with someone at the point of diagnosis to understand their situation, their needs, their goals and the support needs of those that are important to them. (Charity #3)

While online interactions had some benefits, such as reduced travel time and exposure risk, and easier access to specialists, most participants felt that the quality of interactions had suffered, especially for children and young people.

### Getting through this: the importance of support

Managing the hospital alone took a toll on the primary caregiver’s mental health. Caregivers felt they had to ‘stay strong’ for their child, but were often traumatised by their own experiences.

A few people said to me we managed it really well. I really didn’t want to scream and say ‘I didn’t have any other choice!’ I tried my best to navigate it for [child] and the rest of my family. (Caregiver #1)

I couldn't get to hospital without having panic attacks […] Now I struggle to drive to there. I struggle to be outside. (Caregiver #11)

Restrictions prevented many caregivers from accessing the support they felt they needed from family, friends and peers, in person or remotely. A lack of privacy was a key issue for many in feeling comfortable to access any kind of support.

Being able to just see somebody who could support me privately would have been awesome […] I just think the rules made it exceptionally difficult […] how could I pour out my heart about how I was feeling when there was no distance between myself and my [child]? […] you don’t have that safe space to be able to let yourself go. (Caregiver #8)

Caregivers were grateful for the compassionate actions of healthcare staff, with many highlighting the significance of the sense of camaraderie built during a difficult and isolating phase of their journey. For children, many of whom found treatment traumatic, the relationships established during treatment and a supportive and calm environment played a pivotal role.

It was kind of all right because we got to bond a lot […] I knew everyone else was kind of worried, but […] I got a lot of time to myself to think about things, do things I enjoy. (Child #2)

Whenever we were bored, we decided to open our curtains up to each other […] he was a lovely boy. And dad has still got his number on his phone in case anyone wants to phone him and say hello to him, remember how good the memories were. (Child #10)

Caregivers often found it necessary to ‘break the rules’ as the risks to their mental health outweighed the perceived risks of infection.

Sometimes you felt really naughty. I remember at the end of [child]’s treatment, [child] started having seizures and one of the mums came in and I know a nurse had told her to step back and she’s like, ‘no, I’m giving her a hug’ and came in and gave me a hug. And you really need that because you’re all just stood there and no one’s comforting you. (Caregiver #14)

Overall, a huge frustration for caregivers during the pandemic was how they were prevented from accessing their own support systems.

### Supporting the new normal

Returning home after treatment was an important landmark for families. However, during the pandemic, many caregivers experienced an enduring sense of isolation and continued to lack appropriate guidance.

We were so underprepared when we left. We were just given, like a pamphlet, basically. And I look back now and I just think, how on earth were we ever allowed to be sent home with no support? […] It was very difficult, very lonely, very isolating. (Caregiver #20)

The transition to community-based services, which form an integral part of the usual support after discharge, was disrupted by restrictions on home visits, a shift to remote appointments and cancelled outpatient appointments. Temporary closures of ‘non-essential’ services left many families feeling the absence of a ‘safety net’. Remote consultations were rarely experienced as reassuring.

Knowing that normally [a doctor] would have come out [to see the child] and said, ‘yeah, that rash is normal, they get that.’ […] Or ‘maybe we should check this’. That would have put our minds at rest. (Caregiver #12)

Families struggled to access community-based services, particularly where they had not established relationships with those services while in hospital. Navigating complex health and social care systems was experienced as time consuming and frustrating.

It is easy enough to find out about services, but it is harder to know what you should be asking for, what is reasonable, what makes sense for your child—you need the support of someone with experience of brain tumours. (Caregiver #2)

Healthcare professionals commented on the impact of the pandemic on outcomes, in terms of delayed recognition and emergency admissions, and ongoing support for families, in terms of experiences of treatment, and impact on family resilience and mental health. These affected the establishment of a stable ‘new normal’ as disruption was ongoing.

I think one of the big problems we did have was the follow-up appointments, so I think we’ve had a couple of children that had come to be seen and then because of COVID it didn’t get followed up and then they presented later on that were actually really quite poorly […]. I think the outpatient suffered a lot more than the inpatient. (Specialist Nurse #5)

Caregivers were appreciative of strong multidisciplinary and interagency coordination, of having an experienced key worker such as a specialist nurse or clinician, and of proactive guidance and support at key transitions. A specialist multidisciplinary team working across hospital and community operated in one of the study sites, and most caregivers commented that their involvement had been critical in ‘adjusting to the new normal’.

## Discussion

The findings presented in this paper, taken from the qualitative arm of a mixed-methods study, explore the impact of the SARS-CoV-2/COVID-19 pandemic on the diagnosis, management and patient journey for children and young people with a newly diagnosed brain tumour in the UK. The findings highlight the considerable challenges encountered by families and healthcare professionals, which could have had an impact on outcomes. While some issues identified are common to significant diagnoses at any time, the additional challenges of the pandemic on healthcare provision amplified these impacts on families.

Delayed recognition of brain tumours emerged as a clinically and emotionally significant issue, resulting from delayed help-seeking, difficulties in accessing healthcare services and the limitations of remote consultations. Families experienced ongoing challenges after gaining access to treatment, largely as a result of caregivers having to manage hospital time alone. Stakeholders struggled to establish a cohesive and supportive team around the family due to restrictions on their usual practice. Caregivers strived to ensure their child felt safe in hospital, which was challenging when they themselves felt depleted and unable to access the support they needed from family, friends, peers and services. The transition from hospital to home setting accentuated feelings of anxiety and vulnerability as families found themselves alone and without support. In particular, differences between usual care and care during this time were noted. Children and young people are usually supported after discharge by specialist neuro-rehabilitation teams or allied health professionals. Typically at discharge, children and young people have access to a keyworker from the neuro-rehab team or an allied health professional who liaises with community therapy teams. Disruption of community services during the pandemic meant that it was often not possible to form these links, leaving families more isolated. While the pandemic exposed weaknesses in the healthcare system, it also underscored the resilience and adaptability of healthcare professionals and families.

The strengths of this study are that by incorporating multiple perspectives, including those of children and young people, caregivers, clinical staff and charities from different regions of the UK, this study provides a comprehensive understanding of the ongoing challenges linked to the pandemic response. The feedback we received about the interview process was that, despite remembering traumatic memories, it had been cathartic, and participants were keen that their experiences and insights benefit others. Stakeholders’ perspectives were valuable, in that they were able to compare healthcare provision before and during the pandemic in a way that most families were not. Limitations include that participants were self-selecting and that we were unable to recruit any bereaved families, whose perspective may have been particularly valuable in understanding the challenges around late presentation and any relationship to increased risk of mortality. Our reflections on reasons for refusal to participate may be useful to consider for future studies to understand why data are potentially difficult to collect with these groups. As interviews were conducted after the restrictions associated with the pandemic had ended, the retrospective nature of the study posed challenges, particularly for children and young people, in terms of their ability and motivation to recall their experiences.

The findings of this study are consistent with previous research on experiences of the impact of childhood cancer for families,^9 18 19^ and also align with emergent research on how the pandemic disrupted healthcare.^20^,^23^ Evidence suggests that the severity of the impact of COVID-19 infections on paediatric patients with brain tumours was predominately low,^24^,^26^ meaning that the main impact on children was in their experience of delayed diagnosis and experiences of disrupted care. What this study adds are specific insights into the roles of wider services in the delivery of specialist tertiary care. The findings are also likely to be applicable to other complex medical conditions that require a coordinated approach. Reassessing what is considered ‘essential’ service provision may strengthen healthcare collaboration around the child and family. In particular, the role of services like charities in providing support and information was challenged by the delineation of services as essential and non-essential. Our findings suggest that enabling families to access their usual support networks and systems, including peer support, is crucial even in times of severe disruption.

These insights are also relevant to current circumstances as many of the difficulties encountered by families and stakeholders reflect long-standing challenges in healthcare. Building system capacity and effective public health messaging to prompt timely help-seeking are also emphasised. Future research should continue to engage with children and young people directly as their voices are often unheard in clinical research, yet understanding their perspective is vital to improving service provision.

Findings from this study offer practical insights from families and stakeholders to improve the healthcare system during future disruptions. Overall, this study not only sheds light on the challenges faced by families during the pandemic but also identifies recommendations for improving healthcare services to ensure a more comprehensive and effective response in times of crisis.

## supplementary material

10.1136/bmjopen-2024-086118 online supplemental file 1

10.1136/bmjopen-2024-086118 online supplemental file 2

## Data Availability

All data relevant to the study are included in the article or uploaded as supplementary information.

## References

[1] Isba R, Edge R, Auerbach M (2020). COVID-19: Transatlantic Declines in Pediatric Emergency Admissions. Pediatr Emerg Care.

[2] Jalloh I, Smart H, Holland KS (2020). Changes in patterns of activity at a tertiary paediatric neurosurgical centre during the first wave of the 2020 pandemic. *Childs Nerv Syst*.

[3] Isba R, Edge R, Jenner R (2020). Where have all the children gone? Decreases in paediatric emergency department attendances at the start of the COVID-19 pandemic of 2020. Arch Dis Child.

[4] Ronsley R, Bouffet E (2021). COVID-19 in pediatric cancer: Where are the brain tumors?. *Neuro Oncol*.

[5] Office for National Statistics (2017). Cancer registration statistics dataset.

[6] Gatta G, Botta L, Rossi S (2014). Childhood cancer survival in Europe 1999-2007: results of EUROCARE-5-a population-based study. Lancet Oncol.

[7] Wilne S, Collier J, Kennedy C (2012). Progression from first symptom to diagnosis in childhood brain tumours. Eur J Pediatr.

[8] Dixon-Woods M, Findlay M, Young B (2001). Parents’ accounts of obtaining a diagnosis of childhood cancer. Lancet.

[9] Young K, Bowers A, Bradford N (2021). Families’ experiences of child and adolescent brain tumor: A systematic review and synthesis of qualitative research. Psychooncology.

[10] Young K, Miller E, Ekberg S (2020). The Experiences and Healthcare Needs of Families Living With Pediatric Brain Tumor: A Longitudinal Qualitative Study Protocol. Int J Qual Methods.

[11] Jones A (2023). Childhood cancer diagnosis during the COVID-19 pandemic: the parent perspective of the impact on the family.

[12] Sawyer JL, Mishna F, Bouffet E (2023). Bridging the Gap: Exploring the Impact of Hospital Isolation on Peer Relationships Among Children and Adolescents with a Malignant Brain Tumor. Child Adolesc Social Work J.

[13] Braun V, Clarke V (2022). Thematic analysis: a practical guide.

[14] Maxwell J, Mittapalli K, Tashakkori A, Teddlie C (2010). SAGE handbook of mixed methods in social and behavioral research.

[15] Morse JM (2015). Critical Analysis of Strategies for Determining Rigor in Qualitative Inquiry. Qual Health Res.

[16] Malterud K, Siersma VD, Guassora AD (2016). Sample Size in Qualitative Interview Studies: Guided by Information Power. Qual Health Res.

[17] Rabiee P, Sloper P, Beresford B (2005). Doing research with children and young people who do not use speech for communication. Child Soc.

[18] Young K, Cashion C, Hassall T (2023). Supporting families through paediatric brain tumour: Unmet needs and suggestions for change. Psychooncology.

[19] Peikert ML, Inhestern L, Krauth KA (2020). Returning to daily life: a qualitative interview study on parents of childhood cancer survivors in Germany. BMJ Open.

[20] Airth A, Whittle JR, Dimou J (2022). How has the COVID-19 pandemic impacted clinical care and research in Neuro-Oncology?. J Clin Neurosci.

[21] Fisher AP, Patronick J, Gerhardt CA (2021). Impact of COVID-19 on adolescent and emerging adult brain tumor survivors and their parents. Pediatr Blood Cancer.

[22] Sutherland-Foggio MS, Stanek CJ, Buff K (2024). The experiences of families of children with cancer during the COVID-19 pandemic: A qualitative exploration. *Palliat Support Care*.

[23] Vindrola-Padros C, Andrews L, Dowrick A (2020). Perceptions and experiences of healthcare workers during the COVID-19 pandemic in the UK. BMJ Open.

[24] Graetz D, Agulnik A, Ranadive R (2021). Global effect of the COVID-19 pandemic on paediatric cancer care: a cross-sectional study. Lancet Child Adolesc Health.

[25] Mukkada S, Bhakta N, Chantada GL (2021). Global characteristics and outcomes of SARS-CoV-2 infection in children and adolescents with cancer (GRCCC): a cohort study. Lancet Oncol.

[26] Moreira DC, Qaddoumi I, Chen Y (2023). Outcomes of SARS-CoV-2 infection in 126 children and adolescents with central nervous system tumors. Pediatr Blood Cancer.

