# Supplementary material for: Impact of the SARS-CoV-2/COVID-19 pandemic on the patient journeys of those with a newly diagnosed paediatric brain tumour in the UK: a qualitative study
Source: BMJ Open. 2025 Jan 2;15(1):e086118. doi: 10.1136/bmjopen-2024-086118 (PMC11749440; doi:10.1136/bmjopen-2024-086118)
Supplement: online supplemental file 1 [file bmjopen-15-1-s001.pdf]

Supplementary file 1: Interview Schedule for children and young people and parents

Interview topics

1. How has tumour affected you and your family?

2. What was it like finding out about the tumour? Coming to hospital?

3. How did COVID affect things? (masks / one parent / no play / appointments on computer)

4. How could the hospital get things better for other young people and families?

5. Do you have any art / photos / stories you want to share to help people understand your experiences?

6. Is there anything we could do to help other young people tell us about their experiences?

Interview Guide - Parents

Opening
- Introductions / thank you for participating
- Explain purpose of the research
- Overview of interview (areas covered, time, method, recording, breaks)
- Confidentiality
- Consent / sign form / any questions?

1. Before they became unwell, how would you describe your child?

(personality, interests and activities, family, friendships and other important relationships, school, health, what was going on in their life at the time, mood)

2. How did you become aware of the brain tumour (events leading to diagnosis)?

(If we were in a room with participant we could be drawing out a timeline as they speak)

Areas we could prompt if participants do not mention them:

- symptoms pre-diagnosis
- attempts to get help (e.g. GP, optician, A&E, school)
- when and where and by whom the tumour was diagnosed
- your reaction to the diagnosis (thoughts, feelings, behaviours)
- anything you remember as being particularly helpful or unhelpful at the time? (communication with experts, charities, internet forums and sites, friends and family etc)
- prior knowledge / experience of brain tumours / cancer

3. What happened after the tumour was diagnosed?

Areas we could prompt on if participants do not mention them:

- Treatment (neurosurgery, chemotherapy, radiotherapy, etc)
- Timelines (in-patient, out-patient, follow ups etc)

4. As a parent / family, what were your experiences of diagnosis and treatment?

- What was it like for you finding about the tumour?
- thoughts, feelings, behaviours - you and your family

- What was it like for you and your family getting treatment for the tumour?
- Day to day experiences of being in hospital, receiving treatment, attending appointments
- Particular challenges
- Adjustments that you had to make as a family / practicalities
- How you managed / how the family managed (emotion focussed / action focussed etc)
- How did the pandemic affect things for you whilst your child was getting treatment?

- What did you and your family find particularly helpful or unhelpful during this time? (particularly thinking about the pandemic)

- Practicalities e.g. work, staying at hospital, financial support,
- Support from the hospital system - doctors, nurses, play specialists, healthcare professionals, others at hospital

- Support from other patients and people in the hospital
- Support from charities / brain tumour networks / school etc

5. Reflecting on your experiences, what could others learn from them (especially considering future service disruptions)?

- what were the things that services did well, what were the things that they could do differently?

- advice, information, support, practicalities

- what advice would you give to other families who are about to start treatment for a tumour?

6. Debrief

- How was it talking about these things? How are you feeling now?
- Is there anything you want to ask or comment on about the interview?
- Outline what will happen next (e.g. continuing interviews, dissemination plan, etc)
- Are you happy to be contacted with updates on the project?
