# Supplementary material for: Impact of the SARS-CoV-2/COVID-19 pandemic on the patient journeys of those with a newly diagnosed paediatric brain tumour in the UK: a qualitative study
Source: BMJ Open. 2025 Jan 2;15(1):e086118. doi: 10.1136/bmjopen-2024-086118 (PMC11749440; doi:10.1136/bmjopen-2024-086118)
Supplement: online supplemental file 2 [file bmjopen-15-1-s002.docx]

## Supplementary file 2: Interview Schedule for stakeholders

Structured interview guide for stakeholders

(Clinical staff, patients groups & non-commercial third sector)

1. Can you please tell me about your role in this organisation and how long you have been working on this role?
2. What was your day to day routine before the pandemic as part of this role in terms of supporting service user/patients?
3. Looking at the pandemic situation, can you tell me how it has affected your ability to perform this role?
4. How your services were disrupted during the pandemic?
5. How you think this disturbance might have affected your service users/patients?
6. What other issues (from your point of view) have been caused by the pandemic?
7. Looking at the areas (in your services) affected by the pandemic and any lessons

learnt, what needs to be in place during future periods of disruption?

1. Who you think can play a vital role to implement these recommendations and how?
2. Is there anything else you would like to add/clarify?
3. How did you find this experience of reflection?
