# Supplementary material for: Impact of the SARS-CoV-2/COVID-19 pandemic on the patient journeys of those with a newly diagnosed paediatric brain tumour in the UK: a qualitative study
Source: BMJ Open. 2025 Jan 2;15(1):e086118. doi: 10.1136/bmjopen-2024-086118 (PMC11749440; doi:10.1136/bmjopen-2024-086118)
Supplement: Supplementary file 3 [file bmjopen-2024-086118.reviewer_comments.pdf]

PEER REVIEW HISTORY

BMJ Open publishes all reviews undertaken for accepted manuscripts. Reviewers are asked to complete a checklist review form and are provided with free text boxes to elaborate on their assessment. These free text comments are reproduced below.

ARTICLE DETAILS

Title (Provisional)

Impact of the SARS-CoV-2/COVID-19 pandemic on the patient journeys of those with a newly diagnosed paediatric brain tumour in the UK - a qualitative study

Authors

Akhter, Kalsoom; Casson, Roland; Brewster, Liz; Burke, G. A. Amos; Kilday, John-Paul; Macarthur, Donald; Isba, Rachel; Jalloh, Ibrahim

---

VERSION 1 - REVIEW

**Reviewer**	**1**
**Name**	**Rolfe , Melanie**
**Affiliation**	**Queensland University of Technology, School of Nursing**
**Date**	**11-Apr-2024**
**COI**	**No competing interests to declare.**

Overall this is an excellent paper, suitable for publication. If you are making edits you might consider the following, especially in regard to the Discussion section.

Methods

Good use of positioning statement with regards to impact on research.

Recruitment-

Interesting to see the refusal reasons, thank you for including those. Is there opportunity to help these inform future recruitment in the space, for example in articulating that families experiences with the service, whether positive or negative, are useful insights to gather? Or if this was in the recruitment information this would be useful to record here. Was there emotional support offered to participants? What stage in bereavement were families who were invited?

The importance of support

This is a really powerful section, the picture of a nurse trying to deny the mum a hug from her friend is such an exemplar of the issues raised.

Supporting the new normal

More information on the usual support available after discharge would be helpful. What is reported here seems quite usual for many areas.

P8- 'Healthcare professionals commented on...' - This seems like it should be a new section rather than under the 'new normal' heading.

Discussion

More comparison to usual issues faced by families would be useful here, with reference to the literature. Fragmentation of the family unit and difficulty re-integrating after discharge are quite usual- how does this cohort differ?

**Reviewer**	**2**
**Name**	**Moreira, Daniel C**
**Affiliation**	**St Jude Children's Research Hospital**
**Date**	**27-May-2024**
**COI**	**None**

Kalsoom et al present value insight on the impact of the COVID-19 pandemic on patient with CNS tumors and their journey to be diagnosed. The authors should be congratulated on this valuable work.

Major comment:

- A figure that connect the different themes would be valuable to connect the themes that resulted from the qualitative analyses.

- The Discussion needs to be expanded a bit more to contextualize the data. How this is different form other pediatric cancers or how this relates to the pre-pandemic state?

Minor comments:

- I believe some key references are missing in the description of pediatric oncology services during the pandemic (PMID 34454651, PMID 33675698), and specifically for children with CNS tumors (PMID 34415031, PMID 37194498). These will allow to connect the clinical context more robustly,

- Study limitations should be expanded upon. The strengths are mentioned, but not the converse.

VERSION 1 - AUTHOR RESPONSE

Overall this is an excellent paper, suitable for publication. If you are	Thank you for this feedback. We have revised in line with these suggestions.

making edits you might consider the following, especially in regard to the Discussion section.	
Methods<br>Good use of positioning statement with regards to impact on research.	Thank you for this feedback.
Recruitment-<br>Interesting to see the refusal reasons, thank you for including those. Is there opportunity to help these inform future recruitment in the space, for example in articulating that families experiences with the service, whether positive or negative, are useful insights to gather? Or if this was in the recruitment information this would be useful to record here. Was there emotional support offered to participants? What stage in bereavement were families who were invited?	We have added a few sentences to speak to these points in the discussion section, with a view to inform future recruitment.<br><br>In the recruitment section, we have clarified that potential participants were approached if they were diagnosed in the study period and the 12 months prior to the pandemic (i.e. 1st March 2019 to 28th February 2021). This included all outcomes for CYP, including parents who were bereaved.
The importance of support<br>This is a really powerful section, the picture of a nurse trying to deny the mum a hug from her friend is such an exemplar of the issues raised.	Thank you for this feedback – we appreciate your comments on this example.
Supporting the new normal<br>More information on the usual support available after discharge would be helpful. What is reported here seems quite usual for many areas.	We have added more information on support available after discharge.<br><br>This is highlighted in this section, and then clarified in the discussion.
P8- 'Healthcare professionals commented on...' - This seems like it should be a new section rather than under the 'new normal' heading.	We have clarified so that it is more linked to the previous section, showing how healthcare practitioners also recognised the challenges in establishing a new normal for families.
Discussion<br>More comparison to usual issues faced by families would be useful here, with reference to the literature. Fragmentation of the family unit and difficulty re-integrating after discharge are quite usual- how does this cohort differ?	We have added a short section to highlight how this compared with usual issues faced by families in terms of care provided to support re-integration.
Reviewer: 2	

Comments to the Author:<br>Kalsoom et al present value insight on the impact of the COVID-19 pandemic on patient with CNS tumors and their journey to be diagnosed. The authors should be congratulated on this valuable work.	Thank you for these kind words.
Major comment	
- A figure that connect the different themes would be valuable to connect the themes that resulted from the qualitative analyses	A figure has been added.
- The Discussion needs to be expanded a bit more to contextualize the data. How this is different form other pediatric cancers or how this relates to the pre-pandemic state?	Given the word limits, we have added a sentence to clarify -<br><br>"While some issues identified are common to significant diagnoses at any time, the additional challenges of the pandemic on healthcare provision amplified these impacts on families."
Minor comments:<br>- I believe some key references are missing in the description of pediatric oncology services during the pandemic (PMID 34454651, PMID 33675698), and specifically for children with CNS tumors (PMID 34415031, PMID 37194498). These will allow to connect the clinical context more robustly,	Thank you for this comment – we have added these references in to the introduction (reference 4) and discussion (24-26) for context.
- Study limitations should be expanded upon. The strengths are mentioned, but not the converse.	Limitations have been clarified.

VERSION 2 - REVIEW

Reviewer 1
Name Rolfe , Melanie
Affiliation Queensland University of Technology, School of Nursing
Date 25-Nov-2024
COI

Thank you for your consideration of previous comments, these have been suitably addressed.

Reviewer 2
Name Moreira, Daniel C

Affiliation St Jude Children's Research Hospital

Date 27-Nov-2024

COI

The authors have addressed the concerns raised in the first submission.
